# Simple screening model based on electrocardiogram for patients with dilated cardiomyopathy

**DOI:** 10.1097/MD.0000000000032910

**Published:** 2023-02-10

**Authors:** Xiangyu Wang, Qian Zhang, Na Yang, Xishu Wang, Zhiguo Zhang

**Affiliations:** a Department of Cardiology, The First Hospital of Jilin University, Changchun, Jilin Province, China.

**Keywords:** dilated cardiomyopathy, electrocardiogram, nomogram, screening model

## Abstract

Dilated cardiomyopathy (DCM) is one of the most common causes of heart failure. Therefore, screening and early diagnosis of potential DCM patients is beneficial. Electrocardiogram (ECG) can be an inexpensive and easily available screening tool. We aimed to construct a simple screening model for DCM based on electrocardiogram. In this retrospective observational study, we consecutively enrolled 117 DCM patients between July 1, 2016 and July 1, 2021 as the DCM group, while 117 patients hospitalized in the same period with normal echocardiography and ECG were selected as the non-DCM group. Patients were randomly assigned to the training and validation sets at 8:2. ECG parameters of left ventricular related leads were exacted. Logistic regression was performed to evaluate screening indicators of ECG parameters and a nomogram was conducted. The screening ability of the model was evaluated using receiver operating characteristic analysis. Furthermore, the nomogram was assessed using calibration curve and decision curve analysis. Screening indicators included in the nomogram were the amplitude of S wave in V1 and V3 leads, the amplitude of R wave in aVF and V6 leads, and PR interval. The nomogram performed satisfactory discrimination in the training (area under the receiver operating characteristic curve = 0.904) and validation (area under the receiver operating characteristic curve = 0.878) sets and good calibration (Hosmer–Lemeshow *P* = .066). Decision curve analysis demonstrated the model can generate a net benefit of 0.33 when the threshold probability was 0.543. The nomogram based on electrocardiogram is a simple and practical screening tool for potential DCM patients.

## 1. Introduction

Dilated cardiomyopathy (DCM) is a group of cardiac disorders characterized by left ventricular dilatation and systolic dysfunction without severe sufficiently coronary artery diseases or abnormal load conditions, which is one of the most common causes of heart failure.^[1–4]^ First-degree relatives of DCM patients are at a higher risk of developing DCM and can present as sudden cardiac death at first onset.^[5]^ With the increase in the understanding of etiology of DCM, including genetic and non-genetic factors,^[2,6]^ the most common clinical manifestation of DCM including arrhythmias and heart failure can be well explained.^[7,8]^ In spite of the increase in long-term survival of DCM due to optimal pharmacological and non-pharmacological treatment,^[9]^ reducing morbidity and mortality of DCM has still remained a challenge. A timely diagnosis will make a difference in the choice of treatment, and long-term prognosis.

With the rapidly developing cardiovascular imaging technologies, particularly the widespread use of cardiovascular magnetic resonance, we can identify tissue characterization and deepen the phenotype of DCM.^[10]^ Despite the advanced diagnostic technologies, the electrocardiogram (ECG) as the starting point of diagnostic procedures, and an inexpensive and ubiquitously available modality, plays a powerful role in the assessment of patients with DCM. Abnormal ECG, such as left bundle branch block (LBBB) or left ventricular hypertrophy (LVH), will provide “red flag” for the diagnosis of DCM.^[11]^ Applying ECG to population screening of DCM can detect the “red flags” and start the diagnosis and treatment process as early as possible. Nevertheless, the “red flags” in clinical practice required artificial interpretation and lacked sensitivity. This study aimed to correlate DCM with specific ECG parameters rather than ECG patterns, and establish a simple screening model, which can reduce the demand for ECG skills. A simple screening model can be easily applied to an ECG data management system, which especially suits primary healthcare institutions and can improve the efficiency and scope of screening.

## 2. Methods

### 2.1. Study population

Between July 1, 2016 and July 1, 2021, we retrospectively consecutively enrolled 117 DCM patients admitted to the first Hospital of Jilin University as the DCM group, while 117 patients hospitalized in the same period with normal echocardiography and ECG were selected as the non-DCM group. The diagnosis of DCM was based on symptoms, laboratory, and echocardiography. The inclusion criteria for the DCM group were as follows: age ≥ 18 years; Patients underwent 12-lead ECG and echocardiography and the relevant results were stored completely after admission; ECGs were in sinus rhythm; Patients were diagnosed as DCM by echocardiography following the diagnosed criteria that echocardiography showed left ventricular end-diastolic diameter (LVEDD) > 5.0 cm (female) or 5.5cm (male) and left ventricular ejection fraction (LVEF) < 45% according to the guideline protocol of diagnosis of DCM from the British Society of Echocardiography.^[12]^ Patients with normal echocardiography and ECG were selected as the non-DCM group. The exclusion criteria were as follows: ECGs with poor data quality; Patients were diagnosed with ischemic cardiomyopathy for the diameter of main branches of coronary artery obstructive stenosis ≥ 50% verified by coronary CT angiography or coronary angiography; Patients with hypertension, congenital, and valvular or hypertrophic heart disease with severe abnormal load condition sufficient to cause left ventricular dilatation; Long-term excessive drinking history (female > 40g/day; male > 80g/day, lasting for at least 5 years); and Other secondary DCM. Patients were randomly assigned to the training and validation sets at 8:2. This study was approved by the Research Ethics Committee of First Hospital of Jilin University, who waived the requirement for informed consent because this study was a retrospective analysis without any intervention.

### 2.2. Baseline characteristics and ECG data collection

Baseline characteristics included age, gender, body mass index, brain natriuretic peptide level, echocardiographic data, New York heart association class, patterns of ECG, and symptoms and medications. ECG recorded with paper speed = 25 mm/s and calibration = 10 mm/mV. GE Muse ECG data management system (GE Healthcare, Chicago, IL) was used to measure 12-lead ECG parameters. QRS complex parameters were measured using amplitude-based methods.^[13]^ Taking the PR segment as reference baseline, the amplitude of the R wave was the vertical distance from the baseline to the peak of the wave, and the amplitude of the S wave (SA) was the vertical distance from the baseline to the bottom of the wave. PR interval was the duration from the onset of the P wave to the onset of the QRS complex. QRS duration was the time from the starting point of the QRS complex to the end point of the QRS complex. QT interval was the duration from the onset of the QRS complex to the end point of the T wave, and corrected QT interval according to heart rate (QTc) was corrected QT interval according to Bazett formulas. Because DCM is mainly characterized by structural and functional changes in the left ventricular, to simplify the model, we mainly selected left ventricular (LV)-related leads as variables for analysis. The variables were as follows: PR interval, QRS axis, QRS duration, QTc, DIRA, DIIRA, DIIIRA, aVLRA, aVFRA, aVRSA, V1SA, V2SA, V3SA, V5RA, and V6RA. All parameters were checked by 2 experienced physicians.

### 2.3. Statistical analysis

Statistical analyses were performed with IBM SPSS version 26.0 (SPSS Inc., Chicago, IL) and R version 4.1.3. The patients were randomly divided into a training dataset (80%) and a validation dataset (20%) using the “caret” package in R 4.1.3. The training dataset was used to construct the nomogram and the validation dataset was used for external validation. Continuous variables were reported as means ± standard deviations (SDs) or medians (minimum value, maximum value) depending on the data distribution. Categorical variables were reported as frequencies (proportions). Differences between DCM and non-DCM groups were tested by independent sample t-test or Mann–Whitney–Wilcoxon test for continuous variables and chi-square test or Fisher’s exact test for categorical variables. Univariate analyses of ECG variables were performed to identify potential indicators screening DCM. Forward stepwise logistic regression was performed to determine the indicators. Multivariate binary logistic regression analysis with indicators was conducted by the “glm2” package and used to construct the final screening model, which was performed as a nomogram. The area under the receiver operating characteristic curve (AUC) was used to evaluate discrimination and screening ability of the model. The optimal cutoff value was determined using Youden index. Hosmer-Lemeshow test was conducted by the “generalhoslem” package. The decision curve analysis was conducted by the “ggDCA” package and applied to evaluate the clinical effects under the optimal cutoff value of the validation dataset. A 2-sided *P* < .05 was considered statistically significant.

## 3. Results

### 3.1. Baseline characteristics and univariate analyses of ECG parameters

A total of 234 patients including 117 DCM patients and 117 non-DCM patients were enrolled in our study. The baseline characteristics are shown in Table 1. No significant difference in age and gender was observed between the DCM and non-DCM groups. The median age was 51.5 ± 13.1 years in the DCM group and 54.3 ± 11.2 years in the non-DCM group (*P* = .081). 77 (65.8%) were males in the DCM group and 71 (60.7%) were males in the non-DCM group (*P* = .416). The DCM group showed lower LVEF and larger LVEDD, left atrial diameter than the non-DCM group (respectively 29% vs 61%, 68.7mm vs 45.9mm, and 44mm vs 32mm, *P* < .001). The DCM group had higher brain natriuretic peptide level, higher body mass index and worse cardiac function generally, and the most common symptoms in the DCM group were dyspnea and edema while the most common symptoms in the non-DCM group were palpitation and chest pain. The majority of patients in the DCM group accepted “triple therapy” without contraindications. Some patients in the non-DCM group were treated with angiotensin-converting enzyme inhibitors or angiotensin II receptor blocker or angiotensin receptor-neprilysin inhibitor for secondary prevention of coronary heart disease, and some patients were treated with β-blocker as antiarrhythmic drug.

**Table 1:** Baseline characteristics.

	DCM (n = 117)	Non-DCM (n = 117)	*P* value
Age (yr)	51.5 ± 13.1	54.3 ± 11.2	.081
Male sex no. (%)	77 (65.8)	71 (60.7)	.416
BMI (kg/m2)	31.2 ± 6.5	25.7 ± 5.5	<.001
BNP level	646 (95, 2790)	79 (45,595)	<.001
LVEF (%)	29 (22, 36)	61 (54,66)	<.001
LVEDD (mm)	68.7 ± 7.7	45.9 ± 3.1	<.001
LAD (mm)	44 (41, 49)	32 (27,34)	<.001
NYHA Class no. (%)			
I	8 (6.8)	57 (48.7)	<.001
II	23 (19.7)	51 (43.6)	
III	55 (47.0)	9 (7.7)	
IV	31 (26.5)	0 (0)	
Patterns of ECG no. (%)			
LBBB	13 (11.1)	0 (0)	<.001
RBBB	6 (5.1)	0 (0)	<.001
LVH	81 (69.2)	0 (0)	<.001
first-degree AVB	10 (8.5)	0 (0)	<.001
Mobitz type I AVB	2 (1.7)	0 (0)	<.001
Mobitz type II AVB	1 (0.9)	0 (0)	<.001
Symptoms no. (%)			
dyspnea	84 (71.8)	0 (0)	<.001
edema	74 (63.2)	0 (0)	<.001
palpitation	19 (16.2)	43 (36.8)	<.001
chest pain	6 (5.1)	65 (55.6)	<.001
Medications no. (%)			
ACEI/ARB/ARNI	106 (90.6)	59 (50.4)	<.001
β-blocker	94 (80.3)	52 (44.4)	<.001
MRA	104 (88.9)	4 (3.4)	<.001
digoxin	68 (58.1)	0 (0)	<.001
diuretic agent other than MRA	74 (63.2)	0 (0)	<.001

ACEI = angiotensin-converting enzyme inhibitors, ARB = angiotensin II receptor blocker, ARNI = angiotensin receptor-neprilysin inhibitor, AVB = atrioventricular block, BMI = body mass index, BNP = brain natriuretic peptide, DCM = dilated cardiomyopathy, ECG = electrocardiogram, LAD = left atrial diameter, LBBB = left bundle branch block, LVEDD = left ventricular end-diastolic diameter, LVEF = left ventricular ejection fraction, LVH = left ventricular hypertrophy, MRA = mineralocorticoid receptor antagonist, NYHA = New York heart association, RBBB = right bundle branch block.

The training dataset included 94 patients in the DCM group and 97 patients in the non-DCM group. All parameters automatically output by ECG data management system were checked manually and confirmed without error. Univariate analyses identified a number of indicators, including PR interval (173ms vs 156ms, *P* < .001), QRS axis (−2.5°vs 44.0°, *P* < .001), QTc (480ms vs 427ms, *P* < .001), QRS duration (102ms vs 84ms, *P* < .001), DIIRA (0.564mV vs 0.922mV, *P* < .001), DIIIRA (0.188mV vs 0.322mV, *P* = .006), aVLRA (0.512mV vs 0.380mV, *P* = .002), aVFRA (0.292mV vs 0.571mV, *P* < .001), aVRSA (0.000mV vs 0.000mV, *P* < .001), V1SA (1.800mV vs 0.693mV, *P* < .001), V2SA (2.290mV vs 1.090mV, *P* < .001), V3SA (1.570mV vs 0.771mV, *P* < .001), V5RA (1.670mV vs 1.440mV, *P* = .351), and V6RA (1.710mV vs 1.140mV, *P* < .001).

The validation dataset included 23 patients in the DCM group and 20 patients in the non-DCM group. Univariate analyses identified a number of indicators, including QRS axis (−3.0°vs 49.5°, *P* = .038), QTc (475ms vs 436ms, *P* < .001), QRS duration (104ms vs 83ms, *P* < .001), DIIRA (0.429mV vs 0.778mV, *P* = .015), DIIIRA (0.190mV vs 0.391mV, *P* = .006), V1SA (1.620mV vs 0.617mV, *P* < .001), V2SA (2.390mV vs 1.050mV, *P* = .001), and V3SA (1.680mV vs 0.764mV, *P* = .011). Univariate analyses of ECG parameters are shown in Table 2.

**Table 2:** Electrocardiogram parameters and univariate analyses.

Training dataset				Validation dataset		
	DCM (n = 94)	Non-DCM (n = 97)	*P*	DCM (n = 23)	Non-DCM (n = 20)	*P*
PR interval (ms)	173 (120, 262)	156 (53, 364)	<.001	162 (120, 198)	153 (106, 188)	.092
QRS axis (°)	−2.5 (−68.0, 152.0)	44.0 (−52.0, 90.0)	<.001	−3.0 (−44.0, 205.0)	49.5 (−32.0, 86.0)	.038
QRS duration (ms)	102 (78, 192)	84 (63, 405)	<.001	104 (80, 180)	83 (72, 126)	<.001
QTc interval (ms)	480 (406, 597)	427 (371, 4470)	<.001	475 (428, 556)	436 (386, 477)	<.001
DIRA (mV)	0.754 (0.000, 2.350)	0.732 (0.268, 1.570)	.181	0.722 (0.043, 2.150)	0.603 (0.209, 1.370)	.474
DIIRA (mV)	0.564 (0.000, 1.920)	0.922 (0.170, 2.140)	<.001	0.429 (0.000, 1.360)	0.778 (0.351, 1.400)	.015
DIIIRA (mV)	0.188 (0.000, 1.190)	0.322 (0.000, 1.930)	.006	0.190 (0.000, 0.590)	0.391 (0.000, 1.120)	.006
aVLRA (mV)	0.512 (0.019, 1.810)	0.380 (0.00., 1.200)	.002	0.507 (0.063, 1.590)	0.281 (0.000, 1.360)	.077
aVFRA (mV)	0.292 (0.000, 1.500)	0.571 (0.034, 2.030)	<.001	0.229 (0.000, 0.937)	0.630 (0.000, 1.260)	.054
aVRSA (mV)	0.000 (0.000, 1.240)	0.000 (0.000, 1.310)	<.001	0.000 (0.000, 1.680)	0.000 (0.000, 1.040)	.327
V1SA (mV)	1.800 (0.000, 5.810)	0.693 (0.000, 1.900)	<.001	1.620 (0.000, 4.010)	0.617 (0.136, 1.140)	<.001
V2SA (mV)	2.290 (0.000, 5.810)	1.090 (0.087, 2.130)	<.001	2.390 (0.000, 6.010)	1.050 (0.434, 2.020)	.001
V3SA (mV)	1.570 (0.000, 4.450)	0.771 (0.000, 2.620)	<.001	1.680 (0.000, 4.330)	0.764 (0.180, 1.720)	.011
V5RA (mV)	1.670 (0.175, 4.130)	1.440 (0.405, 2.870)	.0351	1.480 (0.024, 4.780)	1.470 (0.742, 2.550)	.980
V6RA (mV)	1.710 (0.029, 4.050)	1.140 (0.302, 2.300)	<.001	1.190 (0.019, 4.070)	1.110 (0.527, 2.500)	.981

DCM = dilated cardiomyopathy, QTc = corrected QT interval according to heart rate, RA = the amplitude of R wave, SA = the amplitude of S wave.

### 3.2. Multivariate binary logistic regression and nomogram construction

**Figure 1.**
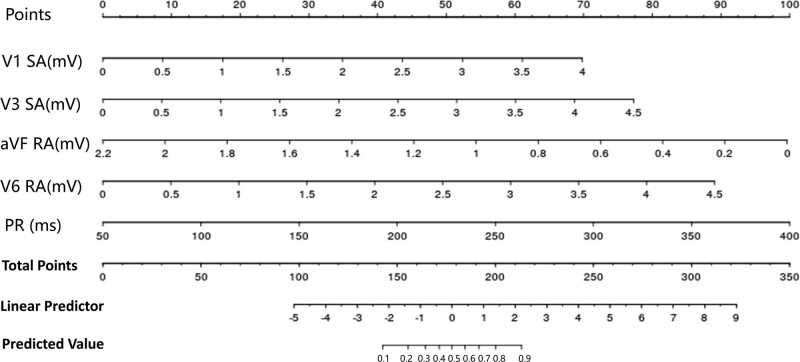
The nomogram of the screening model.

ECG parameters in the training dataset which were found statistically significant in univariate analyses were included in multivariate binary logistic regression using forward LN stepwise regression. After multivariate analysis, V1SA (odds ratio [OR]: 4.145, 95% confidence interval [CI] [2.286, 7.516], *P* < .001), V3SA (OR: 2.597, 95%CI [1.486, 4.539], *P* = .001), aVFRA (OR: 0.072, 95%CI [0.021, 0.249], *P* < .001), V6RA (OR: 2.135, 95%CI [1.137, 4.009], *P* = .018), and PR (OR: 1.022, 95%CI [1.005, 1.039], *P* = .012) remained as independent indicators (Table 3). These results were used to construct the nomogram for screening DCM (Fig. 1).

**Table 3:** Independent screening indicators for DCM in the multivariate logistic analysis.

	β	Odds Ratio(95%CI)	*P*
V1SA	1.4218	4.145 (2.286,7.516)	<.001
V3SA	0.9545	2.597 (1.486,4.539)	.001
aVFRA	−2.6252	0.072 (0.021,0.249)	<.001
V6RA	0.7585	2.135 (1.137,4.009)	.018
itPR	0.0214	1.022 (1.005,1.039)	.012

CI = confidence interval, DCM = dilated cardiomyopathy, RA = the amplitude of R wave, SA = amplitude of S wave.

**Figure 2.**
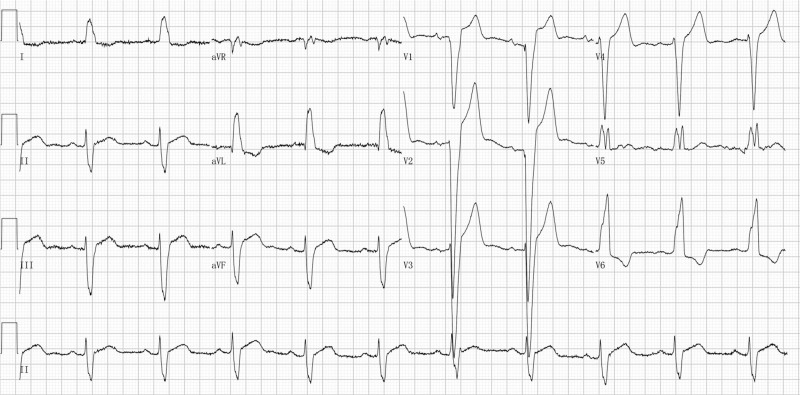
Admission ECG of a patient with dyspnea. ECG = electrocardiogram.

The use of nomogram consists of 3 simple steps. First, the point value of each variable is read on the point scale. Next, add up all the points obtained in the first step as the total point. Finally, the probability of DCM corresponding to the total point of the specific patient is read on the predicted value scale. For example, a patient with dyspnea was admitted to the cardiovascular department of our hospital, and the ECG was examined (paper speed = 25 mm/s and calibration = 10 mm/mV) immediately after admission and showed LBBB, as shown in Figure 2. We measured the ECG parameters included in the nomogram. The PR interval was 190ms, V1SA was 2.626mV, V3SA was 3.579mV, aVFRA was 0.507mV, and V6RA was 1.796mV. The application of the nomogram to this patient showed a total point of 274, with the DCM probability more than 0.9. Bedside echocardiography revealed LVEF of 25% and LVEDD of 69mm. The patient was further diagnostically assessed with coronary angiography when the condition improved, which showed no coronary stenosis. Finally, the patient was diagnosed with DCM after being assessed with cardiovascular magnetic resonance.

### 3.3. Model validation

**Figure 3.**
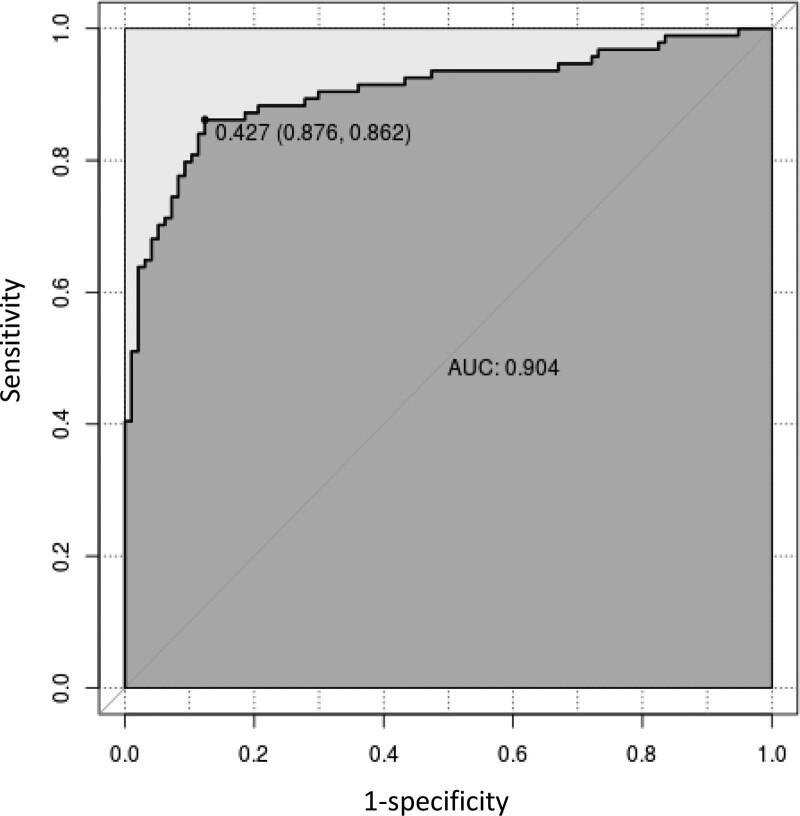
The ROC curve of the training dataset. ROC = receiver operating characteristic.

**Figure 4.**
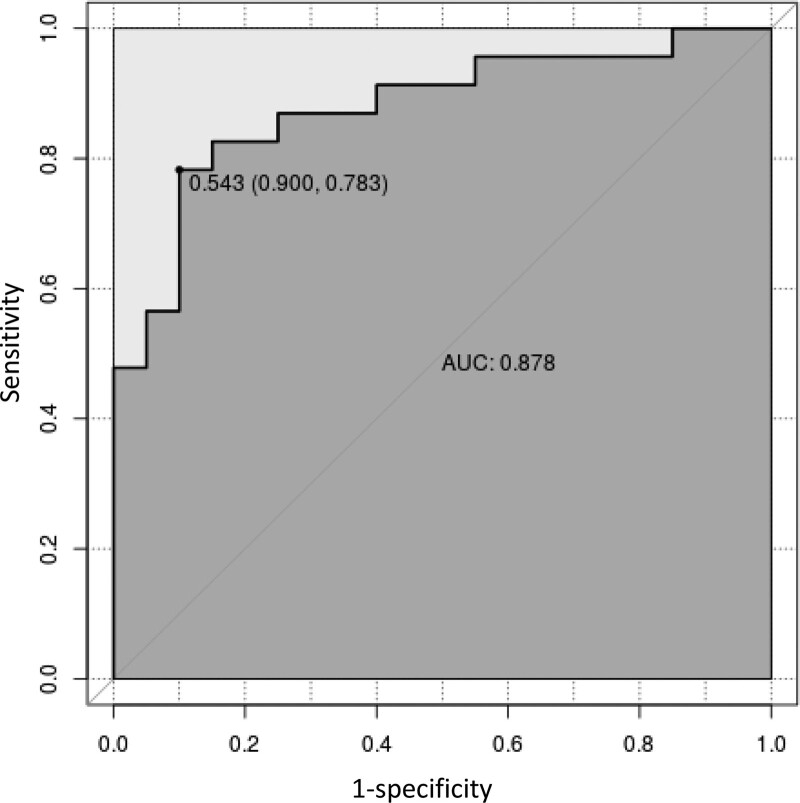
The ROC curve of the validation dataset. ROC = receiver operating characteristic.

**Figure 5.**
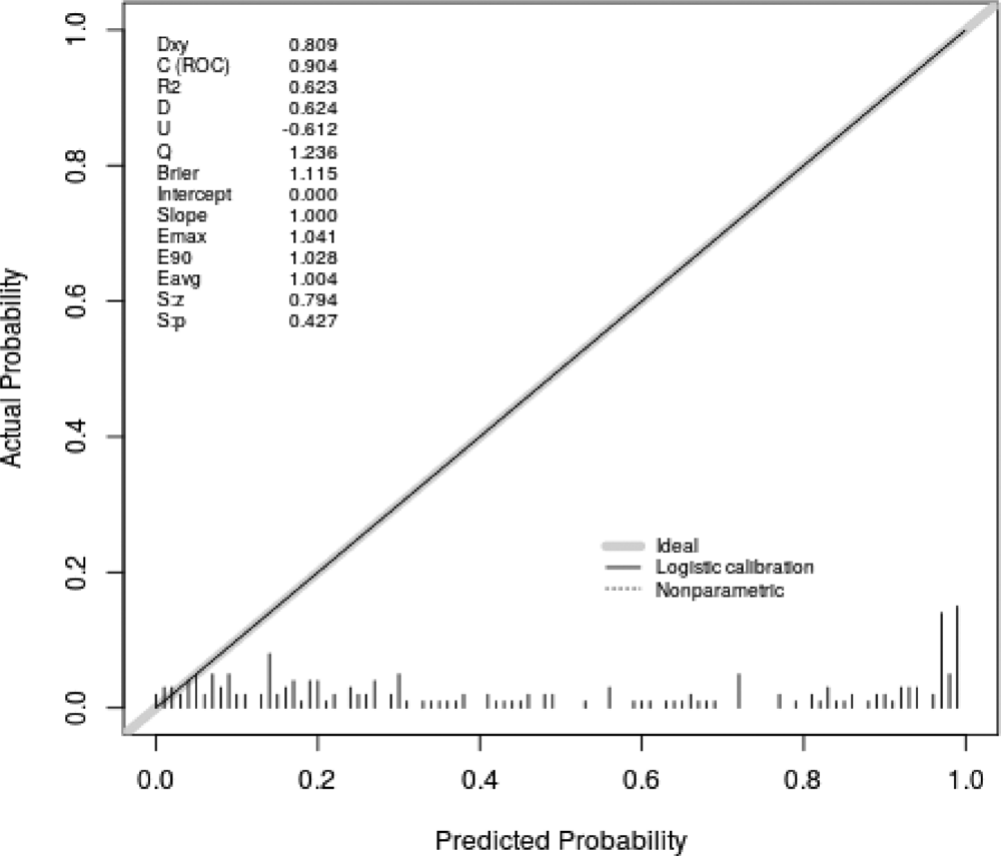
Calibration curve of the training dataset.

**Figure 6.**
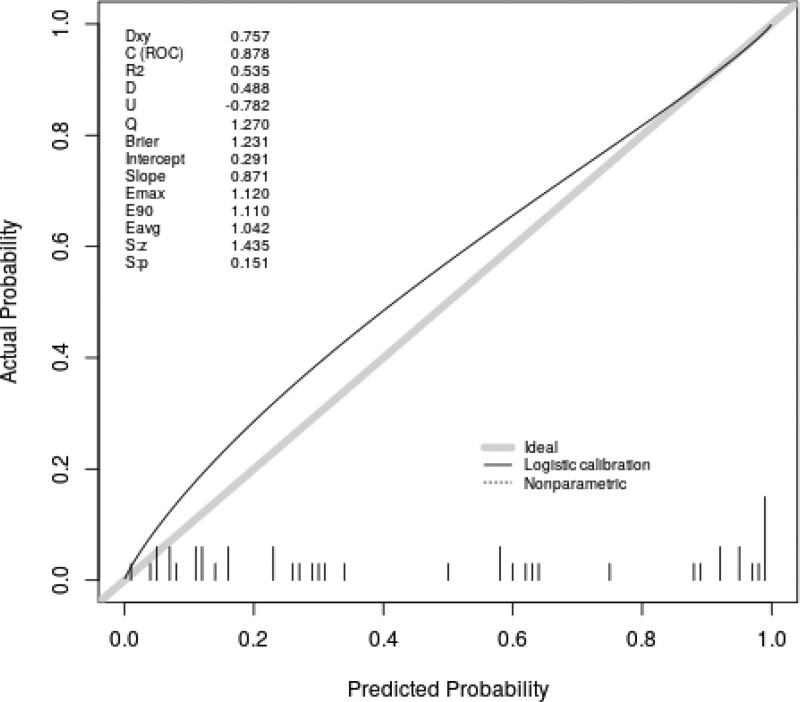
Calibration curve of the validation dataset.

**Figure 7.**
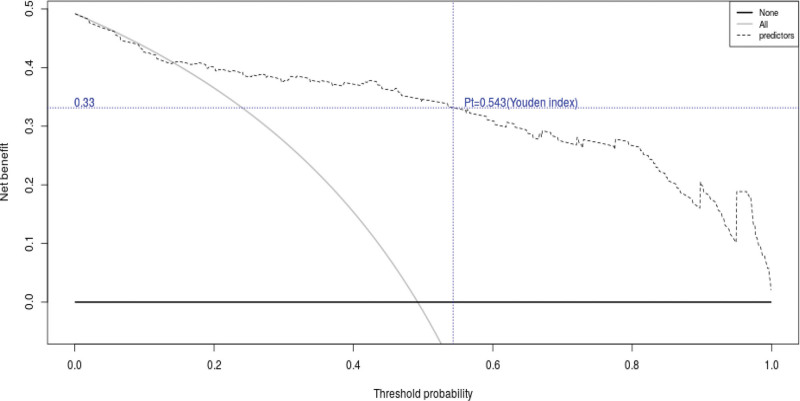
Decision-curve analysis of the validation dataset.

Figure 3 indicated that the AUC value of the nomogram was 0.904 for the training dataset, and the optimal cutoff value was 0.427 with a sensitivity of 86.2% and a specificity of 87.6%. Figure 4 indicated that the AUC value of the nomogram was 0.878 for the validation dataset, and the optimal cutoff value was 0.543 with a sensitivity of 78.3% and a specificity of 90.0%. Figure 5 and Figure 6 displayed the calibration curves of the training and validation sets respectively. The result of Hosmer-Lemeshow test was 14.668 (*P* = .066) in the validation dataset, which indicated a good fitting effect. The decision curve analysis curve was used to illustrate the net benefit of the model for patients from clinical interventions guided by our nomogram. When the threshold value was 0.543 obtained from the Youden index, the model could generate a net benefit of 0.33 (Fig. 7).

## 4. Discussion

Our study constructed a simple screening model for DCM entirely was based on ECG parameters and identified V1SA, V3SA, aVFRA, V6RA, and PR interval as independent screening indicators. ECG, as the first step of the diagnosis of cardiovascular disease, still plays a powerful role in the evaluation of DCM patients. It’s estimated that 80% of patients with DCM have an abnormal ECG.^[14]^ Abnormal ECGs were summarized as stereotypical patterns, such as LVH, LBBB, or first-degree atrioventricular block.^[11]^ However, such interpretation for ECG tracings requires experienced physicians which will increase the costs and burden to the medical system.^[15]^ Previous studies had indicated that a computer-automated ECG screening algorithm can perform well in cardiomyopathy screening.^[16]^ Moreover, our model was constructed from statistical frameworks, therefore, although the procedure of interpretation of ECG was left out for reducing the requirement of medical expertise, our model was easier to explain than models constructed from machine learning without the “black box” phenomena.^[17]^

Historically, many electro cardiologists and clinicians have continuously explored the relationship between ECG and cardiomyopathy. As early as 40 years ago, Goldberger proposed to predict ECG triad in patients with congestive heart failure, including high precordial QRS wave voltage, relatively low limb lead voltage, and poor precordial R wave progression.^[18]^ Finocchiaro summarized the abnormal ECG patterns more specifically, including LVH, LBBB, and first-degree atrioventricular block, etc.^[11]^ The findings are similar to our research. In our study, LVEF of the patients in the DCM group was lower than the non-DCM group, which reflected worse heart function. V1SA was an important clue of DCM, which might be related to the change of cardiac depolarization caused by LV dilatation, LV fibrosis, and LV conduction bundle damage. Although the mean QRS duration in our study did not meet the criteria of LBBB, it was longer than that in the control group. Left bundle branch block was considered to be one of the factors reflecting the risk and progress of DCM.^[2]^ V6RA was also an important indicator. V6RA was higher in DCM patients, which is similar to previous study.^[19]^ In our study, PR interval was longer in the DCM group, which may reflect a trend, although some patients did not meet the diagnostic criteria for first-degree atrioventricular block. Prolonged PR interval reflected the conduction abnormalities, as well as the possible representation of some genetic background, such as ion channel disorder, neuromuscular diseases, or laminopathy.^[6]^ Changes in the PR interval over multiple ECGs may reflect the natural history of DCM. Our study also observed the same ECG patterns as other studies, including anterolateral T-wave inversion, QT prolongation, and left atrial enlargement; however, to simplify the screening model and make it convenient to apply in all levels of healthcare institutions, we constructed the model presented in this paper and selected the screening indicators that were finally included in the model, which showed satisfactory sensitivity and specificity.

Our model is applicable to a wide range of potential DCM patients, especially for first-degree relatives of DCM patients. Familial DCM accounts for approximately 20% - 50% of DCM,^[20]^ therefore, current guidelines recommended that systematic screening for all first-degree relatives of DCM patients, and for those who have causative genetic variants, serial echocardiography was recommended to detect development of disease.^[21]^ However, population-wide echocardiography screening is difficult due to the cost of the test and accessibility. For these reasons, our model as a screening tool has some advantages. Our screening model included quantitative indicators, which were easy to repeat and monitor, and reduced the influence of operator subjective factors and the requirement for medical expertise. In addition, our model can be easily adjusted to fit the multiple screening needs, and adjust the false-positive rate and false-negative rates. Most importantly, in some clinical situation, our model can potentially reduce the need for echocardiography due to the advantage of being inexpensive and readily available, especially in primary healthcare institutions. The results of our model also provide an early signal to further initiate the DCM diagnostic process.

In recent years, the analysis of predictors of ECG for the diagnosis and risk assessment of DCM has been also emerging in endlessly. For example, anterolateral lead T-wave inversion may be a predictor of heart transplantation in patients with DCM, and V2 lead S-wave, III lead R-wave, and anterolateral T-wave inversion may be a predictor of sudden cardiac death in patients with DCM.^[14]^ Nevertheless, the majority of studies were about the predictive value of ECG phenotype rather than parameters for DCM.^[11]^ In our study, we used ECG parameters to predict DCM more intuitively, which is more convenient than the ECG phenotype. And more and more studies have found that the ECG of DCM phenotype caused by different genotypes also has corresponding characteristics.^[22]^ Short PR interval and R/S > 1 in lead V1 may be related to myocardial fibrosis in Duchenne muscular dystrophy.^[23]^ In this study, we did not conduct gene testing, and we hoped that more clinical data could be available to confirm the relationship between patients’ genotype, ECG and DCM in clinical practice. In the future work, we would conduct external validation in independent larger dataset to confirm our model. We also intend to follow up the DCM cohort in present study and evaluate the prognostic value of our model.

### 4.1. Limitations

There are some limitations in our study. First, our study is a retrospective single-center study. The conclusions of our study, especially the cutoff value of the index, need to be further verified in larger datasets. Secondly, the DCM patients we included were mainly hospitalized for collapse of heart failure. This group may have limited benefit and cannot represent all the DCM patients. Thirdly, the patients included in our study were in sinus rhythm, thus the conclusions cannot apply to DCM patients with supraventricular or ventricular arrhythmias. Finally, it is not sufficient to exclude the possibility of DCM only based on our algorithm in clinical, yet further need to investigate other etiology of heart failure practically.

## 5. Conclusion

Our study developed a simple screening model for DCM patients only based on ECG parameters which demonstrated satisfactory performance, and our model is warranted to be validated in more diverse populations.

## Author contributions

**Conceptualization:** Xiangyu Wang, Zhiguo Zhang.

**Data curation:** Xiangyu Wang, Qian Zhang, Na Yang, Xishu Wang.

**Formal analysis:** Xiangyu Wang, Qian Zhang, Na Yang.

**Investigation:** Xiangyu Wang.

**Software:** Xiangyu Wang, Qian Zhang, Na Yang, Xishu Wang.

**Supervision:** Xiangyu Wang.

**Validation:** Xiangyu Wang, Na Yang, Xishu Wang.

**Visualization:** Na Yang.

**Writing – original draft:** Xiangyu Wang, Qian Zhang, Na Yang, Xishu Wang.

**Writing – review & editing:** Xiangyu Wang, Zhiguo Zhang.
